# Is bovine somatotropin an alternative strategy to overcome the detrimental effects of high-gain diets on prepubertal Holstein × Gyr heifers?

**DOI:** 10.1371/journal.pone.0300728

**Published:** 2024-04-29

**Authors:** Anna Luiza Lacerda Sguizzato, Simone Eliza Facioni Guimarães, Giancarlo Magalhães Santos, Erollykens Ferreira Santos, Marcos Inácio Marcondes

**Affiliations:** 1 Department of Animal Science, Universidade Federal de Viçosa, Viçosa, Minas Gerais, Brazil; 2 Cenva Post Graduation, Viçosa, Minas Gerais, Brazil; 3 Department of Animal Science, Washington State University, Pullman, Washington, United States of America; Johnson & Johnson MedTech, UNITED STATES

## Abstract

Feeding high-gain diets and an inadequate energy and protein ratio during pre-puberty may lead to impaired growth and mammary gland development of heifers. Thus, frequent application of bovine somatotropin (**bST**) may prevent future losses in productivity, improve mammary development and animal performance. We aimed to evaluate the effects of bST on digestibility, performance, blood metabolites, mammary gland development, and carcass composition of high-performance prepubertal Holstein × Gyr heifers. Thirty-four Holstein × Gyr heifers with an average initial body weight of 218 ± 49 kg and 14 ± 4 months of age were submitted to an 84-day trial evaluating the effects of no bST or bST injections. Treatments were randomly assigned to each animal within one of the tree blocks. The bST did not influence digestibility or performance parameters. Regarding blood results, IGF1 concentration presented an interaction between treatment and day, where bST heifers had the highest IGF1 concentration. Heifers receiving bST also showed increased ribeye area; however, only an experimental day effect for backfat thickness was observed, with greater accumulation of carcass fat on day 84. Heifers receiving bST had lower pixels/mm² on parenchyma, characteristic of greater parenchymal tissue. Moreover, heifers on bST treatment also had reduced pixels/mm^2^, characteristic of reduced fat pad tissue. Lastly, bST injections did not influence liver and muscle gene expression, nor most genes evaluated in mammary gland tissue, except for *IGFBP3* expression, which was greater for bST heifers. In summary, we confirm the efficacy of bST injections to overcome the detrimental effects of high-gain diets on mammary gland growth and to improve lean carcass gain of prepubertal Holstein × Gyr heifers.

## Introduction

Raising replacement heifers is an essential yet expensive practice on dairy farms. To reduce time in this unproductive phase, feeding diets to achieve increased daily gain rates during pre-puberty has become a widespread management strategy on farms to accelerate growth and hasten puberty [1] and age at first calving, which enables a more rapid profit from heifers’ milk production. However, feeding for high gain, along with an inadequate energy and protein ratio during pre-puberty, may lead to impaired growth and mammary gland development of heifers [2–4].

In Brazil, the majority of the dairy herd is composed of Holstein × Gyr animals, and despite recent efforts to improve energy and protein requirements [5, 6], performance [7], growth [8], and reproduction [9], research is still needed to overcome production flaws such as late puberty [10, 11] and detrimental effects of high gain diets on the mammary gland [4, 12]. In addition, feeding high-gain diets can result in a more significant deposition of subcutaneous backfat thickness in the carcass and increased adipose tissue in the mammary gland of Holstein × Gyr heifers, even when an adequate level of metabolizable protein and energy is fed as a nutritional strategy to reduced damage on mammary growth [4, 12].

The mammary development begins in the fetus [13]. From birth to 2 or 3 months of age, the gland grows at similar rates as the body (isometric), and from around 3 months of age until puberty, or shortly thereafter, mammary growth is allometric [14], when the gland grows at a faster rate compared to the rest of the body. This phase is mainly characterized by restricted duct development and increased udder size by fat pad accumulation, which would later impact secretory tissue development [13, 15, 16]. Thus, knowing that the prepubertal mammary growth will affect the future production capacity of the cow, it is essential to guarantee adequate mammary development, associating nutritional strategies and hormonal pathways [15, 17, 18].

Previous studies have demonstrated a positive correlation between endogenous or exogenous growth hormone (GH), carcass leanness, and mammary growth [2, 19]. GH is secreted from the anterior pituitary and regulates growth and metabolism, activating and promoting the transcription of GH-sensitive genes, such as the IGF1, an essential component of the IGF1/IGFBP3 (insulin-like growth factor binding protein 3) complex. The IGFBP3 is the major IGFBP found in bovine mammary cells and milk, which can inhibit IGF1 action [20]. However, depending on the stimulus, it can exert dependent or independent effects on cell growth, proliferation, and apoptosis [21]. Estrogen, along with the GH, is another hormone responsible for stimulating mammary epithelial cell proliferation [22], resulting in ductal elongation and bifurcation [23].

To our understanding, the sole use of nutritional strategies (as adequate metabolizable protein and energy ratio) is not sufficient to control the negative impacts (fat pad excessive accumulation on mammary stroma) of high gain diets on prepubertal Holstein × Gyr heifers [4, 12]. Thus, using non-nutritional strategies, such as frequent application of bST, may prevent future losses in productivity and promote adequate mammary gland development and animal performance. The bovine somatotropin (**bST**) has the same biological functions as the GH in its natural form [24]. Furthermore, according to the literature, the bST can affect animal homeorhetic status, influence nutrient partitioning, and its use, and impact or not the digestive process [25]. Bovine somatotropin can also stimulate mammary growth by improving circulating levels of IGF1 during pre-puberty [15, 19, 26]. Additionally, it can increase N retention in growing dairy heifers [27] and reduce lipogenesis [25].

Therefore, based on this background and the limiting literature research focused on strategies to overcome the detrimental effects of high gain diets on the development of Holstein × Gyr heifers during pre-puberty, we hypothesized that exogenous bST could stimulate parenchymal tissue growth, decrease adipose tissue deposition in the mammary gland, improve protein deposition on carcass and enhance the performance of prepubertal Holstein × Gyr heifers. Therefore, we aimed to evaluate the effects of bST on digestibility, growth, blood metabolites, mammary gland development, and carcass composition of prepubertal Holstein × Gyr heifers fed high gain diets.

## Material and methods

### Animals, experimental design, and feeding management

This study was carried out in strict accordance with the law n°. 11.794 of October 08^th^, 2008, Decree n°. 6899 of July 15^th^, 2009, and the rules issued by the Brazilian National Council for National Experimentation Control (CONCEA). It was approved by the Ethics Commission on the use of farm animals of Universidade Federal de Viçosa (CEUAP-UFV), protocol n° 0144/2019. Before treatment assignment, all heifers were allocated in *Brachiaria decumbens* paddocks for 24 days, managed in a rotation system. During this period, animals received supplemental feed (5% urea, 30% ground corn, 59% soybean meal, and 6% mineral) every two days in the amount of 700 g/d/heifer and underwent a reproductive assessment to investigate the presence or not of a corpus luteum, indicating the onset of puberty. Consequently, females presenting corpus luteum in one of the ovaries were removed from the trial; additionally, we collected blood from every heifer to evaluate progesterone levels, which, accordingly to Roberts et al. [28], plasma concentration needs to be above 1ng/mL so heifers can be considered pubertal. Therefore, only prepubertal heifers were enrolled in this study. After this period, heifers were transferred to the feedlot and adapted to the experimental diet for 14 days. After that, the 34 heifers were weighed to initiate the trial.

Thirty-four prepubertal Holstein × Gyr heifers with an average initial body weight of 218 ± 49 kg and 14 ± 4 months of age were submitted to an 84-day trial (divided into three experimental periods of 28 days each) to evaluate the use of bST on digestibility, growth, blood metabolites, mammary development, and carcass traits. The 34 heifers were divided into three blocks according to their initial BW (B1 n = 12: 273.6 ± 19.2 kg; B2 n = 12: 214.4 ± 18.3 kg; B3 n = 10: 161 ± 19.7 kg). Moreover, two treatments (**no bST** injections–control or **bST** injections) were randomly assigned to the animals within each block. Thus, the 34 heifers were divided into two blocks of 12 animals each and one block of 10 animals, totaling 17 animals per treatment. Although we initiated the study with 34 heifers, one heifer (Block 2; bST treatment) suffering from a genetic hoof problem had to be removed from the trial at the end of the first period. Therefore, 33 heifers were evaluated in this trial.

Heifers were housed in group pens (six pens with six or five animals each–heifers on the same pen received the same treatment: no bST or bST) with free access to clean water and to a diet formulated to achieve an average daily gain of 1 kg, according to the NRC [29]. The experimental diet consisted of a 65:35 corn silage: concentrate ratio (Table 1). Diet was offered twice daily at 7 a.m. and 3 p.m., and group feed intakes were controlled to allow a 5% leftover (as-fed basis). In addition, every animal in the bST treatment received bST injections (a syringe of 2 g containing 500 mg of recombinant bovine somatotropin ‐ BOOSTIN, Merc Animal Health) subcutaneously in the ischiorectal fossa every 14-day, as recommended by the company, beginning at day 3 and totaling seven administrations. Moreover, to mimic the stress suffered by these heifers, the no bST animals received the same volume of saline injections (sodium chloride, 0.9%), as a placebo, on the same days.

**Table 1:** Composition of ingredients and nutrients in the experimental diet (65:35 silage to concentrate proportion).

Item (g/kg of DM basis)	Roughage	Concentrate	Diet
**Corn silage**			510,25
**Ground corn**		325.43	89.41
**Soybean meal**		618.92	170.05
**Limestone**		31.60	8.68
**Sodium bicarbonate**		12.47	3.42
**Magnesium oxide**		6.24	1.71
**Mineral mix** ^1^		5.34	2.09
**Chemical composition** ^2^			
**DM**	295.22	859.33	492.66
**OM**	947.78	964.93	953.78
**CP**	622.75	300.42	509.93
**EE**	240.67	15.20	161.76
**NDFap**	425.96	127.31	321.43
**NFC**	435.48	521.98	465.76

^1^Composition: calcium, 40 g/kg; phosphorus, 25 g/kg; magnesium, 30 g/kg; sodium, 13 g/kg; potassium, 75 g/kg; sulphur, 10 g/kg; cobalt, 0.8 mg/kg; copper, 63 mg/kg; iodine, 2mg/kg; manganese 185 mg/kg; zinc, 222 mg/kg; selenium 2 mg/kg.

^2^DM = dry matter, OM = organic matter, CP = crude protein, EE = ether extract, NDF = neutral detergent fiber corrected to ashes and protein.

### Digestibility trial, analyses, and calculations

Heifers underwent one digestibility trial at the end of the third experimental period, from day 73 to 80, where they received 10 g/d/heifer of chromium oxide and 15 g/d/heifer of titanium dioxide for six consecutive days (d73 to 79). The chromium oxide was orally infused to determine fecal excretion [30], and the titanium dioxide was offered mixed in the concentrate and divided into two meals to determine concentrate feed intake [31]. Fecal and urine sampling started after five days of marker provision. Three spot samples were collected for feces and urine, each of one on a different day, at 1200h (day 77), 1800h (day 78), and 0600h (day 79). On days 4 to 6, corn silage, concentrate, and refusal samples were also collected.

Feces and silage samples were dried in a ventilated oven at 55°C for 72h or until they were completely dry. Then, feces, silage, and concentrate samples were ground in a Willey mill using 2 and 1-mm sieves [32] to be later analyzed as composite samples. All 1-mm feces, silage, and concentrate samples were analyzed for DM (method 934.01), CP (method 990.13), ether extract (EE; method 2003.05), ash (method 942.05), according to AOAC [33], and NDF corrected for ash and protein contents, according to Detmann et al. [32]. The 2-mm samples were analyzed for undigestible NDF and used as an internal marker to estimate corn silage intake [34]. Moreover, feces were analyzed for chromium oxide and titanium dioxide [32] to estimate fecal excretion and concentrate feed intake, respectively. Digestible energy (DE) and ME were estimated according to the NRC [29], where DE (Mcal/kg) = (5.6 × dCP) + (9.4 × dEE) + (4.2 × dNDF) + (4.2 × dNFC); and ME (Mcal/kg) = 1.01 × DE—0.45.

For spot urine samples (approximately 50 mL), we divided each sample collected at 1200h, 1800h, and 0600h into two samples. Ten mL of urine were diluted into 40 mL of sulfuric acid and stored at -20°C to prevent purine derivative degradation until we performed allantoin analyses according to the technique described by Chen and Gomes [35]. The pure urine sample was used to determine uric acid, urea, and creatinine by the methods described by Kerscher and Ziegenhorn [36], Fujihara et al. [37], and Labtest Diagnóstica S.A, respectively. Total daily urinary excretion was estimated by the method proposed by Chizzotti et al. [38], using the daily creatine excretion for Holstein heifers: CE = 32.2 –(0.0109 × BW), where CE = creatinine excretion and BW = body weight. Purine derivatives excretion was estimated as the sum of daily allantoin and uric acid excretions. Crude microbial protein synthesis was estimated as a function of absorbed purines, calculated from purine derivatives [35].

As agreed among the authors in this study, it is important to state some concerns regarding purine derivatives. It is a useful technique, but it has intrinsic limitations. In their study, Hristov et al. [39] discussed the unequal purine-to-total N ratios in protozoal and bacterial pools associated with the necessity to presume that dietary purines are completely degraded in the rumen [40, 41]. Therefore, according to Hristov et al. [39], calculating absolute changes in microbial protein synthesis based on purine derivatives is not recommended. Nevertheless, it can be used in a controlled experimental trial (as in our study), where the differences in total purine derivatives excretion could indicate a discrepancy in microbial protein synthesis, finally generating concrete interpretations among treatments, as the interpretations observed in our study.

### Growth, average daily gain, body condition score, and feed efficiency estimates

Heifers were weighed on an electronic scale before morning feeding for three consecutive days at the beginning and the end of the trial to evaluate their average daily gain. In addition, withers and hips heights were collected to assess growth on the same days. Intermediary weighting, measurements, and body condition scores were assessed at the beginning of every 28 days to follow animals’ performance and adjust the diet according to their current weight.

### Carcass ultrasound

Heifers were submitted to carcass ultrasound measurements on day 1 and every 28 days to evaluate the rib eye area and backfat thickness. Thus, with the aid of an ultrasound device, we collected images from the *gluteus medius* and *biceps femoris* muscles intercessions and the *longissimus dorsi* [42]. We used an 18-cm linear array ultrasound instrument (Aloka SSD-550V, Aloka Co.), operating at a frequency of 3.5 MHz. Muscle images were recorded and later analyzed using the BioSoft Toolbox^®^ II for Beef software (Biotronics Ins.; [9]).

### Mammary gland ultrasound

Mammary gland ultrasounds were also performed on day 1 and every 28 days. This procedure followed the technique used by Albino et al. [43]. With the aid of a real-time B-mode ultrasound machine equipped with a micro-convex transducer (Mindray DP2200) operating at a frequency of 6 MHz, images were taken of each mammary quarter. Heifers remained standing, and a commercial acoustic gel was applied to the udder quarter before the ultrasound exam. The lubricated micro-convex transducer was positioned at a 45° angle, followed by an image capture of each quarter. Then, we evaluated the pixel value using ImageJ software (NIH) in 8-bit format, collecting three random squares of 4 mm^2^ for parenchyma and 16 mm^2^ for fat pad areas. Next, the pixel value of each mammary quarter was obtained as the mean from the three squares randomly collected near to the parenchyma structures and mammary fat pad of each image. Finally, an average mammary gland pixel value was obtained for both the parenchyma and fat pad. Pixels values were evaluated according to the technique described by Esselburn et al. [44] and Albino et al. [45], where lower pixels values corresponded to more hypoechoic (black) areas, indicating the parenchyma. In contrast, higher pixels values corresponded to more hyperechoic (white) areas, indicating the fat pad.

### Blood sampling and analyses

We collected blood samples to assess IGF1 and insulin levels on heifers submitted to treatments, on day 1 and every 28-d period, and to assess triiodothyronine (**T**_**3**_)_,_ and thyroxine (**T**_**4**_), only at the end of the third period, always before morning feeding. Samples were collected by coccygeal venipuncture with the aid of vacutainer tubes with a gel separator. Tubes were kept on ice until centrifugation (3,000 × *g* at 4°C for 20 min), then serum was pipetted into eppendorf tubes and stored at -20°C until analyses. Analyses of insulin and IGF1 were performed using chemiluminescence immunoassay (Immulite 1000; Siemens Medical Solutions Diagnostics, Los Angeles, USA) [9].

### Liver, muscle, and mammary gland biopsies procedures

To evaluate the effects of bST on the mammary gland, carcass, and liver, we performed biopsies in 18 randomly selected heifers (six of each block–three of each treatment: no bST and bST) on days 85 and 86, right after the last sampling of period three. The first two biopsies performed were on the carcass and liver (day 85). First, heifers were placed in a squeeze chute in a standing position to perform liver and muscle biopsies, followed by cleaning the biopsy site with ethyl alcohol and clipping the hair from a 15 cm^2^ of the area where both incisions were made (liver and muscle). After that, we scrubbed the areas with povidone-iodine and administered anti-inflammatory intravenously via the jugular vein. Before incision, 2% lidocaine hydrochloride was applied subcutaneously for local anesthesia (ribs and *longissimus dorsi*). For the liver biopsy, a one-centimeter incision was made on a line from the tuber coxae to the shoulder point [12, 46], with a scalpel blade followed by introducing a biopsy trocar until the liver was punctured. Then, a syringe was attached to the trocar, and liver tissue was suctioned [46]. For muscle biopsies, an incision of approximately three centimeters was made with a scalpel blade, and, with the aid of a hemostatic forceps, a one-centimeter sample from the *longissimus dorsi* muscle was collected. After tissue collection, the muscle incision was closed following all aseptic techniques. Next, all samples were cleaned with saline solution (0.9% sodium chloride) and stored in liquid nitrogen until quantitative real-time PCR analysis (**qRT-PCR**).

Mammary gland biopsies occurred on day 86. Heifers fasted for 16 hours before the biopsy procedure. Then, each heifer was individually restrained in lateral recumbency and given a dose of general anesthetic (xylazine, 0.5 mL/100 kg of BW). The aseptic procedure followed the same steps described previously for liver and muscle biopsies. A two-centimeter incision was made on the left rear udder on the mid-parenchyma region. After collection, the mammary incision was closed following an aseptic technique, and heifers were released from the restraining position. After the biopsy, heifers were allocated in individual pens, received fresh feed and clean water, and were monitored for five days. Before storage, all samples were cleaned with saline solution (0.9% sodium chloride). Then, samples were kept in liquid nitrogen until qRT-PCR analysis.

### Quantitative real-time PCR analyses

Total muscle RNA was isolated using the Trizol method (Invitrogen and treated with DNase using the RQ1 RNase-Free DNase (Promega. Total RNA was isolated using the PureLink RNA Mini Kit (Thermo Fisher) for liver and mammary gland samples. After that, we performed reverse transcription on muscle, liver, and mammary gland RNA samples using the High-Capacity cDNA Reverse Transcription Kit (Thermo Fisher). Lastly, qRT-PCR was performed in duplicate using the GoTaq PCR Master Mix (Promega) in a QuantStudio 3 thermocycler (Applied Biosystems). Every technique was performed according to the manufacturer’s instructions. The amplification efficiency of internal control and target genes was estimated using four dilutions of cDNA for each tissue evaluated. Amplification conditions for all systems consisted of an initial step at 95°C for 2 minutes, the second step of 40 cycles at 95°C for 15 s, and a final extension step at 60°C for one minute. After the amplification cycles, an additional gradient step from 60°C to 95°C was used to obtain a melting curve.

The ΔCt method was used to estimate the expression of each gene (target Ct–internal control Ct), where Ct represents the PCR cycle number of cDNA amplification above the threshold level. Moreover, gene expression differences were estimated using the -2^ΔCt^ method [47]. Target genes evaluated in the present study were: *mTOR* and *AMPK* for muscle; *IGF1* and *GHR* for liver; and *IGF1*, *IGF1R*, *IGFBP3*, *FASN*, and *ESR1* for mammary gland. Primer pairs for internal control and target genes are presented in Table 2, according to their identification sequences from the GenBank database.

**Table 2:** Gene name, primer pair sequence, annealing temperature, and amplification efficiency of each target gene.

Genes^1^	Accession number^2^	Primer sequence (5´- 3´)	Amplicon, bp
***IGF1***	NM_001077828.1	Forward: AGCAGTCTTCCAACCCAATTA	103
		Reverse: ACAGGGCCAGATAGAAGAGA	
***GHR***	NM_176608.1	Forward: CCTCAACTGGACTCTACTGAAC	112
		Reverse: CCAGGATTATCCATCCCATCTT	
***mTOR***	XM_002694043.6	Forward: AAGGAGAAGGAACGGACA	
		Reverse: CCAGCACACGAGGTAAATAG	
***AMPK***	NM_001109802.2	Forward: AGTTGCCTACCACCTCAT	
		Reverse: GTGGTGATCGTCGAGAAAC	
***IGF1R***	NM_001244612.1	Forward: GTATGGAGGAGCCAAGCTAAA	123
		Reverse: GTCTTGGCCTGAACGTAGAA	
***IGFBP3***	NM_174556.1	Forward: CTCCACTTCATGCCTTAGCA	120
		Reverse: GACAGGGCGTTCTTCTTCTT	
***FASN***	XM_005220997.2	Forward: CAACAAAACTGGTGCTCACG	122
		Reverse: ATCAACTCTGAGGGGCTGAA	
***ER1***	Connor et al., 2005^3^	Forward: TTGCTGGCTACTTCGTCTC	148
		Reverse: GGTGGATGTGGTCCTTCTC	
***RSP15A***	NM_001037443.2	Forward: GGAGTGATCAGCCCTAGATTTG	108
		Reverse: AGCTGAGGTTGTCAGTACAATG	
***18S***	NR_036642.1	Forward: GCCGCTAGAGGTGAAATTCT	129
		Reverse: TCGGAACTACGACGGTATCT	
***GAPDH***	NM_001034034.2	Forward: GATGCTGGTGCTGAGTATGT	113
		Reverse: GCAGAAGGTGCAGAGATGAT	

^1^Genes: insulin-like growth factor 1 (*IGF1*), growth hormone receptor (*GHR*), (*mTOR*), (*AMPK*), insulin-like growth factor 1 receptor (*IGF1R*), insulin-like growth factor-binding protein 3 (*IGFBP3*), fatty acid synthase (FASN), estrogen receptor alpha (*ER1*), ribosomal protein S15A (*RSP15A*), 18S ribosomal RNA (*18S*), glyceraldehyde 3-phosphate dehydrogenase (*GAPDH*).

^2^GenBank.

^3^Connor et al. [48]

### Statistical analyses

All variables were analyzed using PROC GLIMMIX of SAS (Statistical Analysis System, 9.4 version) in a randomized complete block design, using initial BW as blocking criteria. Feed intake, digestibility, average daily gain, serum T_3_, T_4,_ and gene data did not present repeated measures; thus, they were analyzed according to the following model:

$${\text{Y}}_{\text{ijk}}=\mu +{\text{T}}_{\text{i}}+{\text{B}}_{\text{j}}+\varepsilon_{\text{ijk}}$$ (1)

μ = mean; T_i_ = fixed effect of treatment i; B_j_ = random effect of block j; and ε_ijk_ = random error with mean 0 and variance σ2, variance among animal measurements.

Mammary gland and carcass ultrasound, body condition score, growth, and serum IGF1 and insulin data were evaluated along the experimental period (day 1, 28, 56, and 84); therefore, period measurements were included as repeated measures in the model as follows:

$${\text{Y}}_{\text{ijklm}}=\mu +{\text{T}}_{\text{i}}+\delta_{\text{ij}}+{\text{B}}_{\text{k}}+{\text{P}}_{\text{l}}+{\left(\text{T}\times \text{P}\right)}_{\text{il}}+\varepsilon_{\text{ijklm}}$$ (2)

μ = mean; T_i_ = fixed effect of treatment i; δ_ij_ = random error with mean 0 and variance σ2, the variance among animals within treatment equal to the covariance among repeated measures among animals; B_k_ = random effect of block k; P_l_ = fixed effect of period l; (T x P)_il_ = random effect of interaction between treatment i and period l; and ε_ijklm_ = random error with mean 0 and variance σ2, variance among animal measurements. Measurements on day 1 were included as covariates for carcass characteristics, body condition score, and growth models.

Eight variance-covariance structures (AR, CS, FA, UN, TOEP, VC, ARH1, TOEPH) were tested and the one presenting the best fit based on the Akaike information criterion was used. Body condition score was used as a co-variable for itself, growth, and carcass ultrasound variables. In addition, due to the unbalanced design (one heifer removed from the trial), degrees of freedom were corrected using the Kenward-Rodger approximation. The main effects of bST and days were discussed separately in the absence of interactions. For all analyses, significance was declared when *P* ≤ 0.05.

## Results

### Feed intake and digestibility of heifers

According to the digestibility trial conducted on days 73 to 80, the bST did not influence feed intake or digestibility (*P* ≥ 0.142; Table 3), metabolizable energy, metabolizable protein, and their relations (*P* ≥ 0.216), or microbial efficiency and nitrogen balance (*P* ≥ 0.103).

**Table 3:** Feed intake and diet digestibility of Holstein x Gyr heifers submitted to control or bST treatment^1^.

	Treatment		SEM	*P*-value^3^
	no bST	bST		Treatment
**Feed intake** ^2^				
**DM, kg/d**	7.873	7.711	0.709	0.783
**CP, kg/d**	1.160	1.140	0.100	0.802
**NDFap, kg/d**	2.420	2.253	0.172	0.308
**EE, kg/d**	0.150	0.139	0.010	0.261
**NFC, kg/d**	3.932	3.539	0.293	0.221
**OM, kg/d**	7.511	7.357	0.676	0.783
**DMI/BW, g/kg of BW**	25.943	24,741	1.390	0.469
**NDFap/BW, kg/kg of BW**	7.536	6.8858	0.753	0.396
**RDP intake, g/d**	572.790	510.890	36.609	0.190
**RUP intake, g/d**	584.240	627.960	116.650	0.532
**MEI, Mcal/kg**	20.975	18.738	1.509	0.186
**MPI, g/kg**	835.550	830.090	82.181	0.926
**MPI/BW, g/kg of BW**	2.744	2.656	0.124	0.614
**MEI/BW Mcal/kg of BW**	66.137	61.046	4.446	0.216
**MP:ME, g/Mcal**	40.630	41.866	1.321	0.513
**Digestibility** ^2^				
**DM, g/kg**	649.230	639.950	8.629	0.453
**CP, g/kg**	675.150	666.230	8.685	0.473
**NDFap, g/kg**	445.460	451.270	17.817	0.788
**EE, g/kg**	831.030	804.120	14.759	0.208
**NFC, g/kg**	849.310	834.930	6.7479	0.142
**OM, g/kg**	689.08	683.400	8.567	0.643
**Nitrogen balance**				
**Microbial efficiency**	109.170	101.950	13.235	0.465
**Excreted, g/100 kg of BW**	44.395	40.499	2.797	0.103
**Retained, g/100 kg of BW**	15.670	18.230	1.746	0.308

^1^ Feed intake and digestibility were assessed during the last experimental period.

^2^NDFap = neutral detergent fiber free of ashes and protein, EE = ether extract, MEI = metabolizable energy intake, MPI = metabolizable protein intake.

^3^ *P*-values indicate treatment effects (*P* ≤ 0.05).

### Growth, average daily gain, body condition score, and feed efficiency estimates

**Fig 1:**
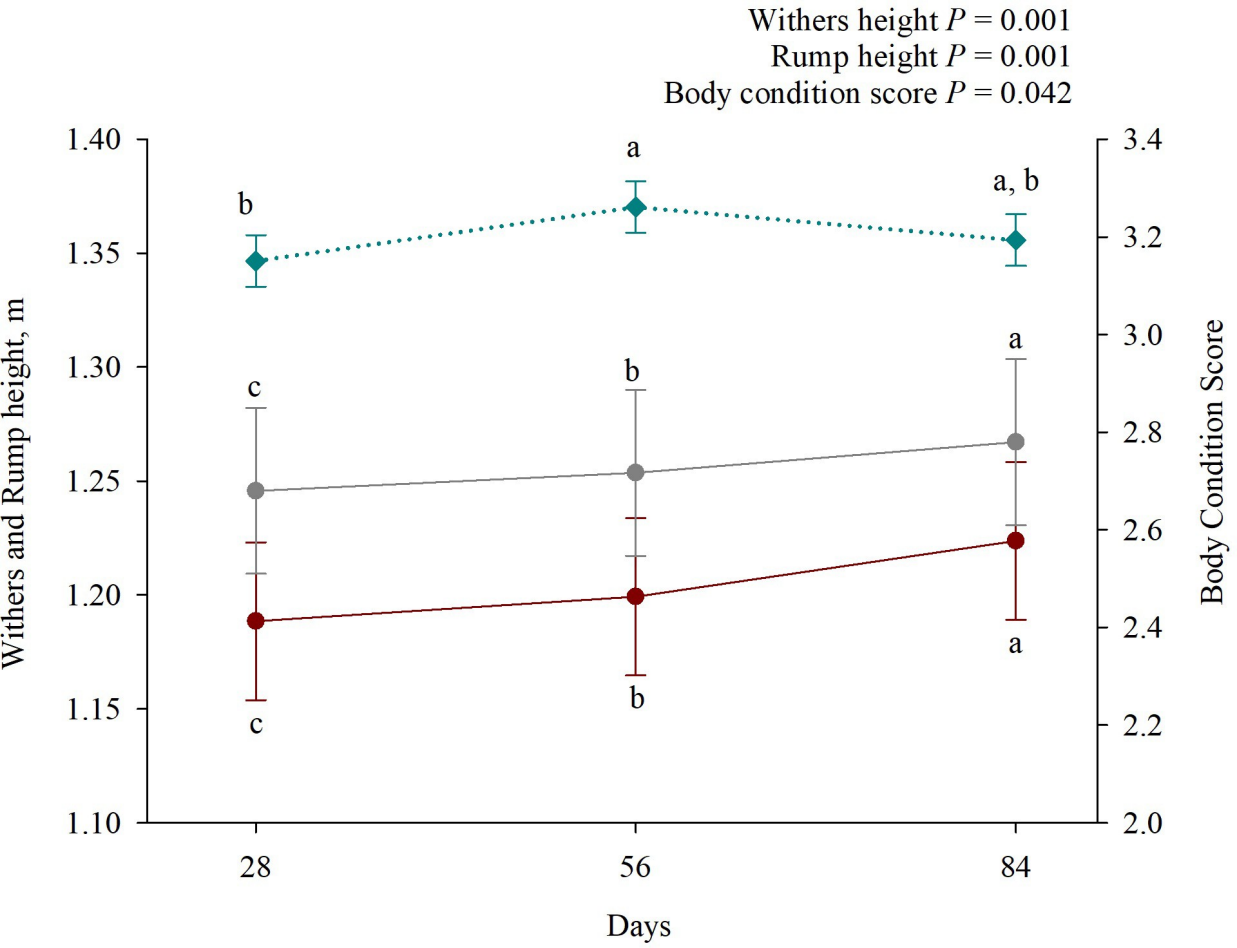
Experimental day effect on Holstein × Gyr heifers’ withers and rump height and body condition score. Closed red circles represent withers height, closed grey circles ‐ rump height, and blue diamond ‐ body condition scores. Statistical differences were considered when *P* ‐ value was ≤ 0.05.

Heifers receiving bST injections had the same final body weight, average daily gain, and withers and rump height as heifers that did not receive this treatment (*P* ≥ 0.266; Table 4), but animals grew during the experimental period, both for wither and rump height (*P* = 0.001; Fig 1). Regarding body condition score, it also increased along the days evaluated (*P* = 0.042; Fig 1), presenting a trend between treatment and experimental day interaction (*P* = 0.081). Moreover, there was no statistical difference in feed efficiency between treatments (*P* = 0.574).

**Table 4:** Performance of Holstein x Gyr heifers submitted to control or bST treatment.

	Treatment		Day			SEM	*P*-value^2^		
	no bST	bST	28	56	84		T	D	T × D
**Performance** ^1^									
**Initial BW, kg**	209.86	229.66				12.360	0.266		
**Final BW, kg**	305.19	314.58				37.131	0.323		
**ADG, kg/d**	1.12	1.15				37.132	0.726		
**Withers height, m**	1.20	1.21	1.19^c^	1.20^b^	1.22^a^	0.035	0.436	0.001	0.922
**Rump height, m**	1.25	1.26	1.24^c^	1.25^b^	1.27^a^	0.037	0.750	0.001	0.291
**BCS**	3.23	3.17	3.15^c^	3.26^a^	3.19^b^	0.056	0.482	0.042	0.081
**Feed Efficiency (kg ADG/kg DMI)**	14.51	15.16				0.811	0.574		

^a-b^ Means without common superscript letters in the same row between 2 treatments over 3 periods are significantly different (*P* ≤ 0.05).

^1^BW = body weight, ADG = average daily gain, BCS = body condition score, and DMI = dry matter intake.

^*2*^*P*-values indicate treatment (T), experimental day (D), and treatment by experimental day interaction (T × D) effects.

### Serum IGF1, insulin and thyroid hormones

**Fig 2:**
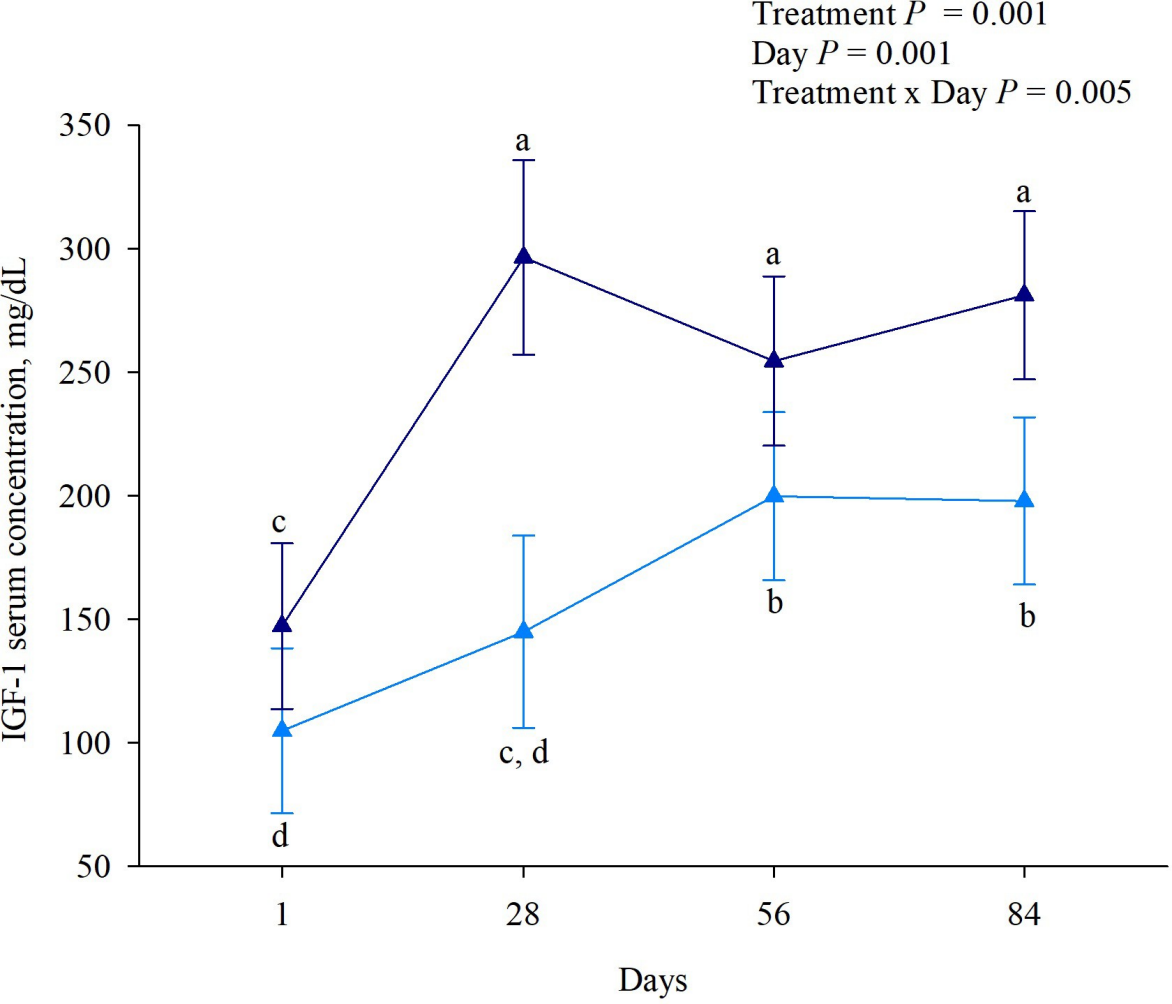
Treatment and day interaction on IGF1 serum concentration of Holstein × Gyr Heifers. Dark blue triangles represent bST treatment, and light blue triangles represent no bST treatment. Differences were considered when *P* ‐ value was ≤ 0.05.

We observed no interaction between the experimental day and treatment for insulin (*P* = 0.332; Table 5). However, there was an experimental day effect (*P* = 0.025), with increased insulin concentration on day 84 and a tendency to increase it on no bST treatment of heifers (*P* = 0.079). Regarding IGF1 concentration, we observed an interaction between treatment and experimental day (*P* = 0.005; Fig 2), where heifers on the bST treatment had the highest concentration of IGF1 regardless of the day. Heifers on the no bST treatment showed greater IGF1 serum concentrations on days 56 and 84, but their overall concentration was continuously below the bST treatment.

**Table 5:** Blood metabolites concentration of Holstein x Gyr heifers submitted to control or bST treatment.

	Treatment		Day					*P*-value^2^		
	no bST	bST	1	28	56	84	SEM	T	D	T × D
**Blood metabolites** ^1^										
**Insulin, μUI/dL**	0.26	0.17	0.14^c^	0.25^ab^	0.18^bc^	0.29^a^	0.036	0.079	0.025	0.332
**IGF1, ng/mL**	161.87	244.88	126.04^b^	220.73^a^	227.17^a^	239.57^a^	32.997	0.001	0.001	0.005
**T**_**3**_ **, ng/mL**	1.10	1.28					0.065	0.064		
**T**_**4**_ **, ng/mL**	4.49	5.76					0.386	0.002		

^a-c^ Means without common superscript letters in the same row between 2 treatments over 3 periods are significantly different (*P* ≤ 0.05).

^1^T_3_ = triiodothyronine, T_4_ = thyroxine.

^*2*^*P*-values indicate treatment (T), experimental day (D), and treatment by experimental day interaction (T × D) effects.

T_3_ and T_4_ serum concentrations were evaluated only on day 84. We observed a trend of increased T_3_ serum concentration on bST animals (*P* = 0.064) and higher T_4_ concentration for these same heifers (*P* = 0.002).

### Carcass ultrasound

**Fig 3:**
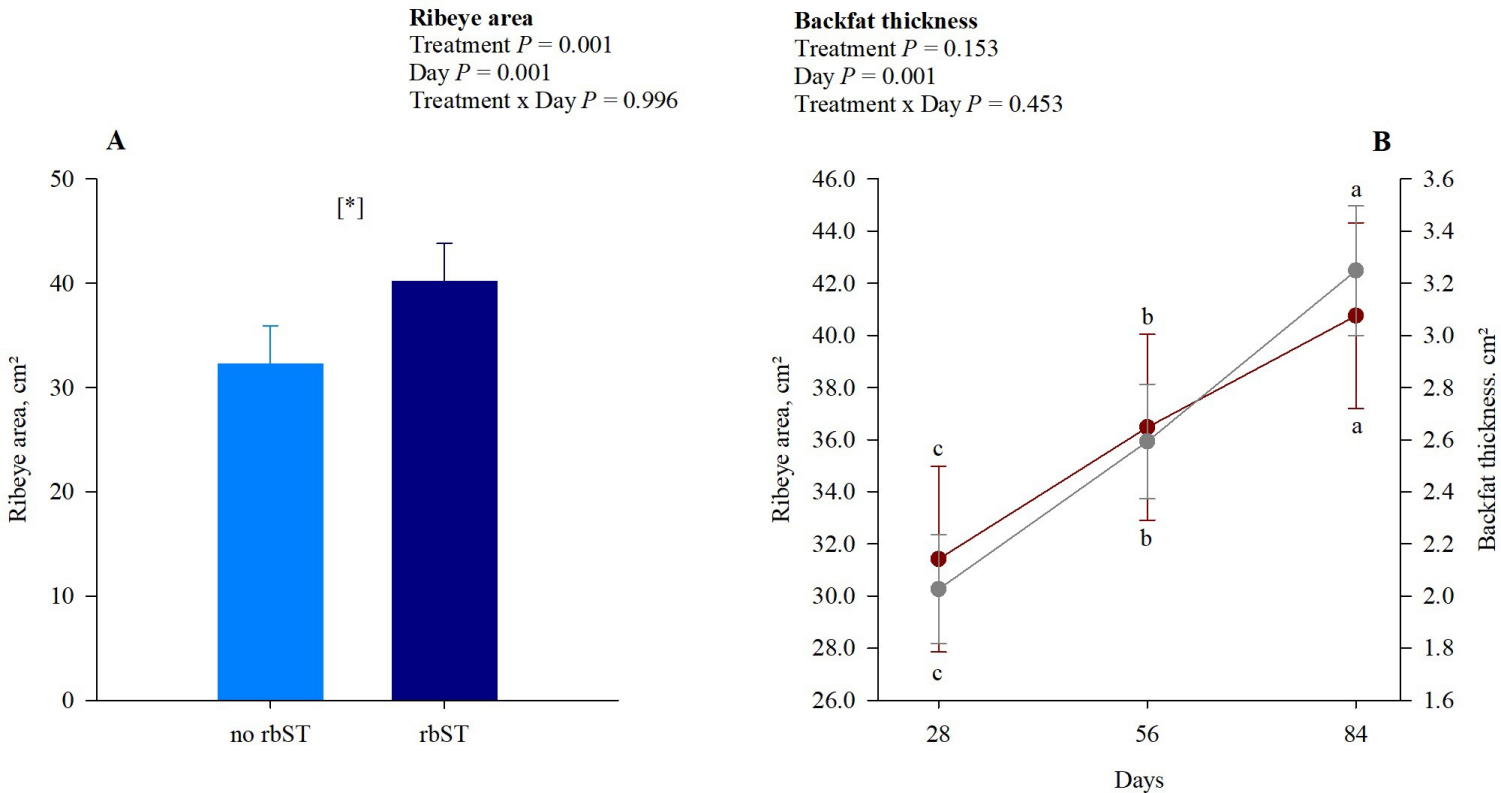
Representation of carcass ultrasound results. A ‐ Carcass ribeye area between treatments. B ‐ Carcass ribeye area and backfat thickness among days. Red circles represent the ribeye area, and grey circles represent backfat thickness. Statistical differences were considered when *P*-value was ≤ 0.05.

Heifers receiving the bST injections showed an increased ribeye area compared to the no bST treatment, around 25% greater deposition of lean tissue (*P* = 0.001; Fig 3). This increase in the rib eye area (cm²) was also observed along the days evaluated, with the greatest area observed on experimental day 84 (40.76; *P* = 0.001). On the other hand, we observed only an experimental day effect for backfat thickness (cm^2^), with a more significant accumulation of carcass fat on experimental day 84 (*P* = 0.001). No interactions were observed between treatment and experimental day (*P* ≥ 0.453).

### Mammary gland ultrasound

**Fig 4:**
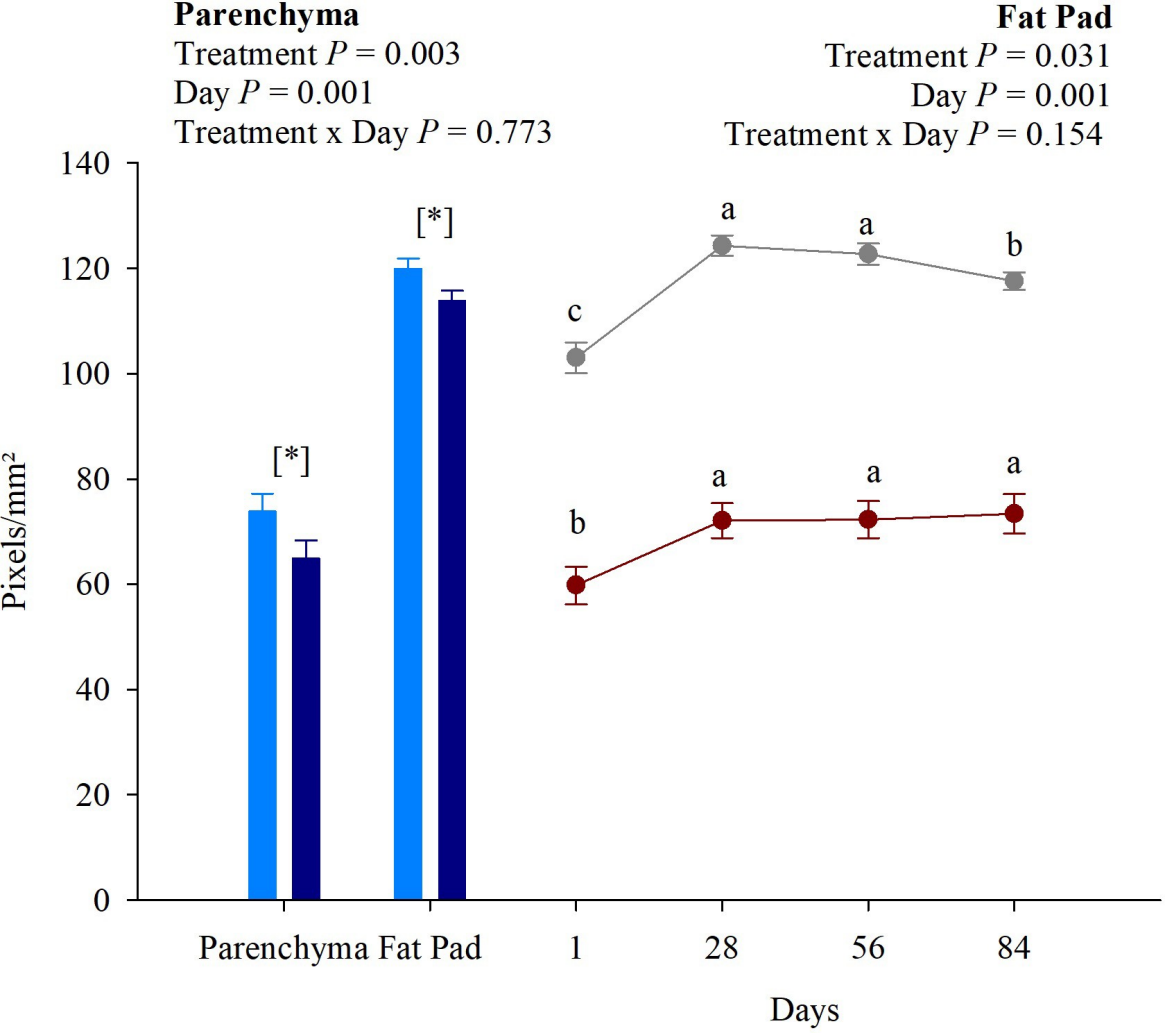
Representation of mammary gland ultrasound results. Light blue columns represent no bST treatment, and dark blue columns represent bST treatment in the parenchyma and fat pad areas. Red circles represent parenchyma, and grey circles represent fat pad areas among the experimental days. Statistical differences were considered when *P* ≤ 0.05.

Heifers receiving bST injections had lower pixels/mm^2^ on parenchyma when compared with no bST treatment (*P* = 0.003), meaning a less brightening color, such as black, which is characteristic of parenchymal tissue. According to Esselburn et al. [44], a more hypoechoic (black) area is indicative of parenchyma, whereas more hyperechoic (white) areas are the fat pad. In addition, we also observed an experimental day effect on mammary parenchyma, with the lowest pixels/mm^2^ on day 1 (59.79; *P* = 0.001), which increased on the first 28 experimental days and remained constant until the end of the trial (Fig 4). Regarding fat pad tissue, heifers on bST treatment also had reduced pixels/mm^2^ (*P* = 0.031), regardless of time. Animals presented the lowest fad pad pixels/mm^2^ on experimental day 1, followed by experimental day 84, and the highest values were observed on experimental days 28 and 56. Moreover, no interactions were observed between treatment and experimental day for parenchyma and fat pad (*P* = 0.773 and *P* = 0.154, respectively).

### Liver, muscle, and mammary gland genes expressions

The application of bST did not influence liver and muscle gene expression (*P* ≥ 0.323 and *P* ≥ 0.442, respectively; Table 6). In addition, bST injections did not affect most genes evaluated in mammary gland tissue (*IGF1*, *IGF1R*, *FAS*, *ER1*; *P ≥* 0.207), except for *IGFBP3* expression, which was greater for no bST treatment when compared to heifers receiving bST injections (0.28 vs. 0.15; *P* = 0.023).

**Table 6:** Liver, muscle, and mammary gland gene expression of Holstein x Gyr heifers.

	Treatment		SEM	*P*-value^2^
Genes^1^	no bST	bST		Treatment
**Liver**				
***IGF1***	0.144	0.136	0.017	0.739
***GHR***	0.518	0.439	0.055	0.323
**Muscle**				
***mTOR***	0.0001	0.0001	0.00001	0.442
***AMPK***	0.0002	0.0002	0.00005	0.803
**Mammary gland**				
***IGF1***	0.017	0.009	0.004	0.207
***IGF1R***	0.019	0.027	0.006	0.337
***IGFBP3***	0.276	0.151	0.035	0.023
***FASN***	0.005	0.006	0.0016	0.706
***ER1***	0.059	0.048	0.015	0.605

^1^Insulin-like growth factor 1 (*IGF1*), growth hormone receptor (*GHR*), (*mTOR*), (*AMPK*), insulin-like growth factor 1 receptor (*IGF1R*), insulin-like growth factor-binding protein 3 (*IGFBP3*), fatty acid synthase (FASN), estrogen receptor alpha (*ER1)*.

^*2*^*P*-values indicate treatment effects (*P* ≤ 0.05).

## Discussion

The use of bST is a strategy used on farms to improve animals’ performance, milk production, and mammary development. Nevertheless, very few studies have evaluated bST effects on Holstein × Gyr heifers, a crossbred of substantial economic impact on the dairy system of tropical countries. Thus, we observed the necessity of understanding the effects of this non-nutritional strategy on prepubertal Holstein × Gyr heifers. To confirm the veracity of prepubertal status of heifers used in this trial, data in S1 Table presents progesterone levels on the first day of the trial, which were lower than 1ng/mL, and reproductive assessment results. Additional information regarding intake, digestibility, performance, ultrasounds, and gene expression can be found in S2 Table.

The bST is known to have an homeorhetic effect on animals, which can alter nutrient partitioning and redirect its use after intestinal absorption. However, the bST effect on the digestive process is minimal [25, 27]. Our results follow Eisemann et al. [49] and Crooker et al. [27], who evaluated the effects of bST on dietary intake and digestibility of Hereford and Holstein heifers, respectively. They observed no statistical difference in nutrient intake or digestibility with the use of bST injections. Additionally, these authors reported a significant decrease in urinary nitrogen excretion, which was not observed in our study. Likewise, Nascimento et al. [50] did not observe differences in nutrient intake. Still, heifers receiving bST injections every 14 days had greater crude protein and organic matter apparent digestibility than control or single-dose treatment. Contradicting our findings and the ones reported in the literature, Gandra et al. [51] observed increased dry matter intake as a percentage of BW of Holstein heifers receiving bST injections.

Although voluntary feed intake increases in lactating cows due to enhanced energetic demand for milk production [25, 52], the same biological response is not observed in growing animals. In our study, heifers had a dry matter intake (7.85 kg of dry matter) close to the feed intake predicted by NRC [29] and Silva et al. [53] for heifers with an average body weight of 314 kg (7.20 kg of dry matter). Moreover, evaluating the performance according to the new NASEM [54] software, we observed that the initial diet formulated for heifers to achieve 1 kg/d actually predicted 1.2 kg/d of energy allowed for growth (frame and reserves). Thus, the results in our study regarding average daily gain and, consequently, final body weight indicate an improvement in prediction equations used in the 8^th^ version of NASEM [54].

Lima et al. [55], also evaluating the effects of bST on Holstein × Gyr heifers, observed no somatotropin effects on average daily gain and final BW. However, in another study, Fudimoto et al. [56] reported an increased final BW of Holstein × Gyr heifers treated with bST every 30 days. Moreover, there is a considerable difference when comparing our results with studies conducted with pure Holstein heifers, as in Radcliff et al. [19], who reported increased average daily gain and higher withers height of pubertal Holstein heifers receiving bST injections. Gandra et al. [51] also showed that bST injections could improve thoracic perimeter, length, rump width, and BW, but bST only affected average daily gain for 60 days; and Moallem et al. [57] confirmed that bST injections enhanced Holstein heifers performance from 90 to 314 days of age, increasing BW and hip height, but no effect was observed on heifers from 314 until 644 days of age. In our study, no effect of bST on withers and rump height was observed. Albeit the proven bST effect on promoting lipolysis and improving animals’ lean carcass gain, mainly characterized by the local action of IGF1, none of these outcomes were observed in our study [19, 25, 58].

To better understand the limiting response of bST on animals, we can relate it to the saturation of GH receptors on these heifers. Campos [59] evaluated increasing doses of bST on milk production of crossbred Holstein × Gyr cows; however, no differences between doses were observed. The author described the lack of results as a saturation effect of GH receptors on crossbred animals due to their reduced genetic merit for milk production compared to the Holstein breed. According to Bauman [25], animals with reduced genetic merit have decreased circulating endogenous GH levels. Therefore, in Campos [59] study, Holstein × Gyr cows would need a lower level or exogenous bST to achieve their maximum production, consequently caused by the saturation of GH receptors. Despite the distinct physiological phase (growing animals vs. lactating cows), we speculate that the same explanation [59] can be attributed to our study. Therefore, the Holstein × Gyr heifers evaluated in our study could present saturation of GH receptors, leading to the limiting performance response. However, in this case, it would not be attributed to lower genetic merit but to an increased feeding level. It is well known that under high feeding levels, GH concentration is reduced as much as its receptors [60–62]. Thus, GH receptors’ saturation may have occurred primarily due to the increased feeding level heifers were subjected to, causing the somatotropic axis uncoupling. Thus, this primary response did not allow the exogenous administration of bST to exert its full effect on animals’ performance, occasioning the minor effects of bST on the heifer’s growth and development.

The limiting response of bST on growth and development may also be assimilated with the ones obtained with serum concentration of blood parameters. The insulin, a pancreatic hormone highly correlated with feeding level, acts to promote the storage of metabolites in peripheral tissues such as skeletal muscles and adipose tissue. However, its role in hepatic tissues for the ruminant species seems less important once glucose uptake by the liver is minimal in this specie [63]. Therefore, in our study, the increase in serum insulin over the days could be attributed to the daily feed intake of animals, which grew according to the heifers’ body weight. However, since we did not perform a digestibility trial every 28 d, we cannot confidently explain the higher insulin serum concentration on day 28 of the experimental period, although it is possibly a response to the greater feed intake of nutrients at that specific sampling period.

Regarding the tendency of increased insulin serum concentration observed on no bST heifers, our results go against the literature, which suggests a possible interaction between this hormone and the bST, wherein the exogenous or endogenous growth hormone would allow an insulinemic state in periparturient dairy cows, with a less pronounced effect on growing heifers [64–66]. Additionally, Hall et al. [67] observed increased insulin serum concentrations in heifers receiving bST injections. The bST may not have caused an insulin-resistant state on our heifers, but it could have impaired, or reduced insulin secretion on bST animals once feed intake variables remained the same between treatments.

Growth hormone and IGF1 concentrations at birth are considerably lower than at puberty. The coupling of the somatotropic axis during puberty promotes an increase in GH release from the pituitary gland and greater expression of its receptors, resulting in higher production and secretion of IGF1 [68]. Moreover, some authors [26, 69, 70] reported an increased IGF1 serum concentration in heifers treated with exogenous somatotropin. Therefore, our results concerning IGF1 serum concentration are in accordance with literature findings, confirming a higher concentration of this hormone on bST-treated heifers and according to the proximity of days towards puberty.

Since thyroid hormones are known to be related to metabolic activities, homeostasis processes, and animal responses to nutritional level, reproductive and immunological status [71], they can act synergistically with somatotropin enhancing growth [72]. According to Root et al. [72], the GH influences the peripheral metabolism of thyroid hormones, enhancing T_4_ degradation and its conversion to T_3_. This pattern of T_3_ serum concentration agrees with the tendency observed on bST heifers. However, to our knowledge, no studies suggest an explanation for the increased T_4_ levels in bST-treated heifers; neither it was possible to associate T_4_ results with an increased metabolic rate of these animals. Therefore, despite the limiting results on average daily gain or feed efficiency, the greater serum concentration of IGF1, T_3_ and T_4_ likely acted to improve carcass muscle deposition and mammary growth on bST heifers.

The 25% increase in the ribeye area of bST heifers can be linked to greater IGF1 concentrations observed for these animals. Growth hormone improves liver production and release of IGF1 in the blood; then, this insulin growth factor will act on target tissues, in this case, on the carcass, stimulating lean gain and protein accretion [25, 73]. Radcliff et al. [19] observed improved carcass weight and protein deposition in heifers receiving bST injections. On the other hand, bST treatment was inefficient in reducing carcass adipose tissue deposition, as observed by Moallem et al. [74]. In our present study, we noticed around a 60% increase in backfat thickness area along the days, without difference between treatments, which agrees with body condition score results, although there was a greater body condition score on day 56 of the experimental period.

Prepubertal Holstein × Gyr heifers can present higher fat deposition on the carcass when fed high-gain diets [4], which can explain the 60% increase in backfat thickness. Nevertheless, our results indicate that, to control their homeorhetic status, heifers prioritized their metabolism for lean carcass and mainly mammary gland growth (discussion in the next section), which agrees with the accelerated growth rate of these tissues during this physiological stage, pre-puberty [14], despite reducing backfat thickness deposition. Therefore, based on previous studies, our assumption that exogenous GH would improve lean gain was confirmed, suggesting the efficacy of bST injections to improve carcass growth in Holstein × Gyr heifers.

Focusing on the mammary gland, the effects of somatotropin on its growth during pre-puberty are extensively discussed in the literature, as much as the essential actions of estrogen [62, 75]. The pituitary somatotropin promotes mammary growth mediating the activation of the GH-IGF axis, where part of GH signaling occurs through the mammary stromal cells and via paracrine production of IGF1 [76, 77]. However, when feeding diets for elevated growth rates (1 kg/day), GH levels decrease, uncoupling the GH-IGF axis [15]. Thus, the exogenous administration of bST can improve mammary gland growth when the serum concentration of GH is reduced by increasing the percentage of protein, the amount of RNA, and total parenchyma on the mammary gland of prepubertal heifers [19, 76].

**Fig 5:**
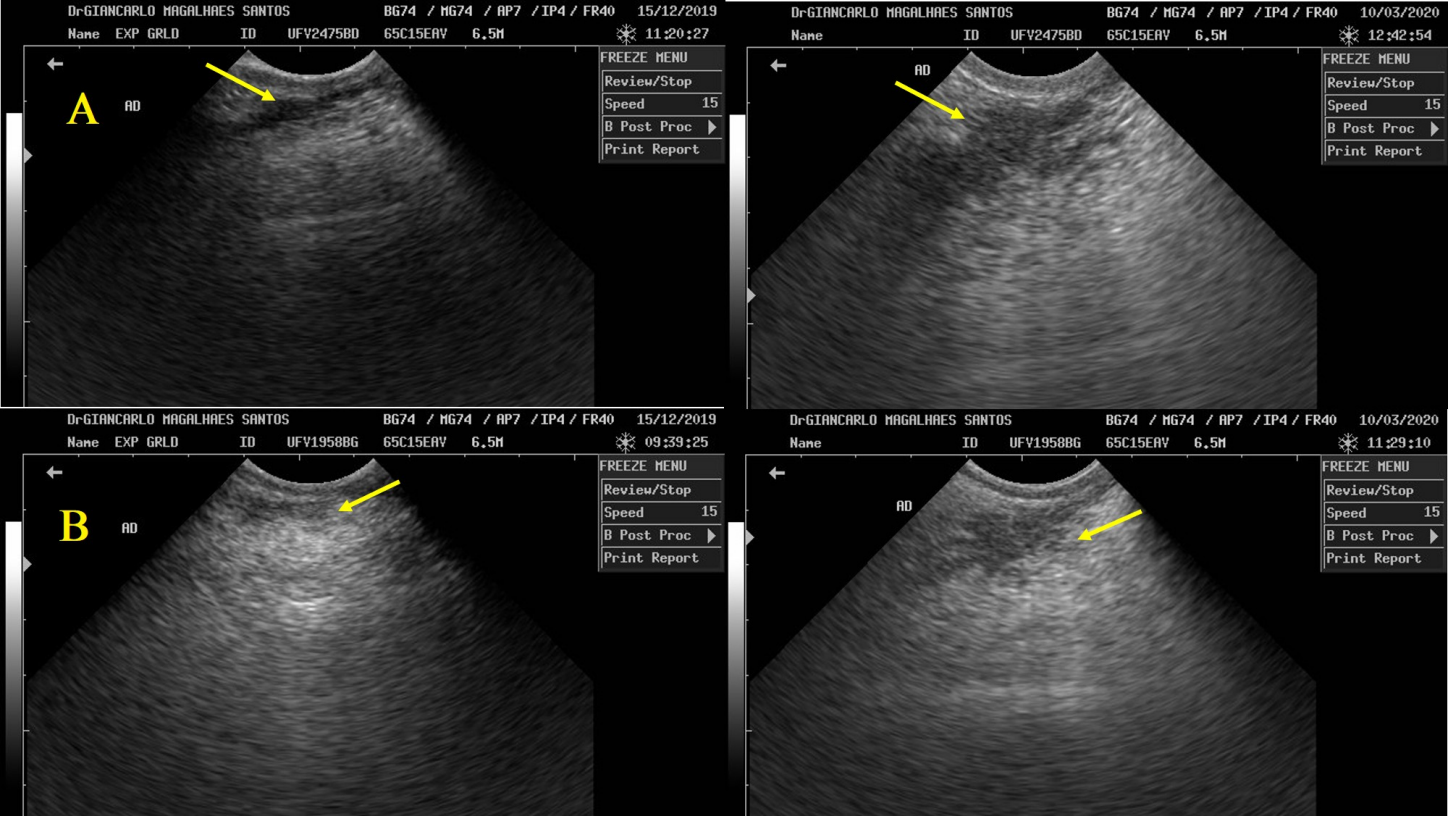
Increase in the mammary parenchyma tissue of Holstein x Gyr heifers (indicated by yellow arrows). A ‐ Heifer in the bST treatment, days 1 and 84. B ‐ Heifer in no bST treatment, days 1 and 84.

The technique study by Albino et al. [43] and validated with Holstein × Gyr heifers allowed us to observe a more remarkable parenchymal growth on bST-treated heifers through mammary gland ultrasound. The lower pixels/mm^2^ found in the parenchyma of bST animals indicate enhanced ductal growth compared to heifers with no bST treatment. According to Albino et al. [43], the ultrasound images do not distinguish between epithelial duct tissue and duct lumens of parenchyma; however, it assures a difference in tissue growth. Sejrsen et al. [78] and Radcliff et al. [19] also observed differences in the amount of parenchymal content of the mammary gland, either by protein percentage or by RNA and total parenchyma. Moreover, we also observed an increase in parenchyma pixels/mm^2^ from day 1 to 28; thus, considering that the parenchyma is an epithelial tissue surrounded by intra- and inter-lobular stroma, this result suggests the elongation and growth of ductal tissue which penetrates the mammary stroma (Fig 5).

Regarding fat pad tissue, we suspect that the sharp increase observed in the first 28 days of the trial occurred due to some residual compensatory gain. Before the beginning of the experimental period, heifers were not fed to achieve high growth rates, they were fed on a supplemented pasture system for one month ‐ daily free access to a well-managed *Brachiaria decumbens* and approximately 1 kg of concentrate supplemented three times a week. Therefore, their feed intake was likely limited, as well as nutrient partitioning and availability to mammary development. However, with a balanced diet and exogenous growth hormone provision, the mammary gland had the ideal nutritional and hormonal environment to develop, as indicated by greater circulating levels of IGF1 and increased parenchymal tissue.

The mammary fad pad has an essential role in the proliferation of parenchyma and synthesizes growth factors that have mitogenic actions [79]. However, it can also be detrimental to mammary development, especially in Holstein × Gyr heifers [4], which do not seem to respond to increased feeding levels as pure Holsteins heifers, even when adequate MP:ME ratios are fed [45]. Therefore, to our understanding, the bST treatment stimulated parenchyma tissue growth by enhancing serum concentration of IGF1 and promoting duct elongation towards mammary stroma, which minimized the detrimental effects of high gain diets on the mammary gland of prepubertal Holstein × Gyr heifers.

Gene expression analyses can also explain some of the responses discussed previously. The absence of changes in liver *GHR* expression due to bST treatment follows Radcliff et al. [26] findings. According to these authors, bST injections do not alter the *GHR* mRNA expression of non-producing animals as they do on lactating cows. This response could be attributed to distinct metabolic or endocrine changes driven by animals’ homeorhetic status, frequency of bST injections, or hepatic GH bindings peak around puberty [26]. In addition, we can also suggest a decreased GHR for Holstein × Gyr heifers due to their inferior genetic merit for milk production compared to purebred Holstein heifers [25, 59]. Moreover, in our study, bST injections increased only serum IGF1 concentration but not its expression in the liver. Although Radcliff et al. [26] did not observe differences in *GHR* expression on bST-injected heifers, they found an increased expression of IGF1 mRNA. Therefore, considering that bST administration exclusively improved serum IGF1 concentration, we assumed that the extra IGF1 produced by the liver acted on target tissues, such as muscle and mammary gland, enhancing their growth rates.

The *mTOR* regulates cellular machinery by exporting growth-promoting mRNA and enhancing protein synthesis [80]. In rats, Hayashi and Proud [81] confirmed the process of protein synthesis from *GH* action towards the *mTOR* signaling pathway, which occurs possibly through activation of the PI 3-kinase and the *PKB/Akt*. Moreover, the *AMPK* activation suppresses the *mTOR* pathway during low energetic status reducing protein synthesis [82]. In a recent study evaluating the effects of bST on beef heifers, Hergenreder et al. [83] observed an increase in *AMPKα* expression, which was related to reduced marbling score and greater fiber cross-sectional area of bST-treated heifers. However, despite the outcomes presented in the literature, bST treatment did not affect *mTOR* or *AMPK* expression which could be expected considering that *mTOR* activity hardly changes. Thus, we performed a posteriori analysis to investigate *mTOR* activity, which would provide us with a better understanding of the metabolism within lean tissue deposition in heifers treated with bST. The target antibodies for this analysis were procured from Cell Signalling (9452S – *4E-BP1* antibody; 9451S –*phospho-4E-BP1* antibody; 2211S –*phospho-S6 ribossomal protein* antibody; and 2217S –*S6 ribossomal protein*). Prior to commencing the immunoblotting analysis, we undertook a thorough examination of the samples’ protein integrity, which was optimum, given their extended storage duration exceeding two years. Thus, fifteen micrograms of protein for each sample were meticulously loaded onto a 15% SDS Page gel for separation, following the methodology proposed by Martins et al. [84], with certain adaptations. Primary antibodies were diluted to ratios of 1:50, 1:100, and 1:300. Similarly, the secondary antibody (*HRP anti-rabbit*; BA1054; Booster Bio) was diluted to a ratio of 1:1000, and the revelation was accomplished using DAB (3,3′-diaminobenzidine).

The proteins marked with DAB successfully identified the beta-actin band protein (sample’s integrity antibody), but not the targeted proteins (*S6* or *4E-BP1*). Despite the negative response to DAB revealing process, we observed a high rate of success on each step of the immunoblotting analysis, leading us to the conclusion that, possibly, the primary antibodies chosen to be assessed did not actively react with bovine tissue. Therefore, our efforts to better understand the metabolism of lean tissue deposition in Holstein × Gyr heifers treated with bST reinforced the need for a deeper investigation of *mTOR* activity in this particular experimental condition.

Regarding the genes assessed in the mammary gland tissue, the bST treatment impacted their expression to a lower extent than we expected. Plath-Gabler et al. [85] observed considerable expression of *IGF1* on the mammary gland of virgin heifers (18 months of age), indicating an intense proliferative role for this hormone at this phase. Albino et al. [4] and Weller et al. [12] observed an enhanced expression of *IGF1R* and *FASN*, respectively, in Holstein × Gyr heifers fed high-gain diets compared to maintenance animals. Moreover, according to Weller et al. [12], the expression of *FASN*, one of the genes involved in adipose tissue synthesis, can be controlled by dietary and hormonal characteristics. Nevertheless, none of these genes had altered expression according to our treatments.

The major response for mammary gene expression was observed for *IGFBP3*, which was lower for heifers receiving bST injections. Lew et al. [86] evaluated the gene expression profile of Holstein heifers’ mammary gland while animals were fed different diets and received bST injections. They observed an altered expression of fifty-three tissue-developing genes, up-regulating thirty-four proliferative and two anti-proliferative genes, only due to bST treatment. Moreover, among the seventeen genes downregulated by bST treatment, six were classified as anti-proliferative, such as the *IGFBP3*. The *IGFBP3* is the major IGFBP found in bovine mammary cells and milk, which can interfere with *IGF1* action, as demonstrated by its exogenous application [20]. However, Leibowitz and Cohick [21] observed that *IGFBP3* could exert dependent or independent effects on cell growth, proliferation, and apoptosis. Thus, dependent on the stimulus, the ternary complex formed among the *IGF*, *IGFBP3*, and an acid-labile unit can hinder *IGF* translocation to the target tissue, impairing tissue growth or development [12].

Similar to our finding, Berry et al. [70] also evaluated the bST effects on heifers’ mammary gland and observed a reduction in *IGFBP3* expression in their mammary parenchyma. In addition, Berry et al. [70] also found an enhanced serum IGF1 concentration in heifers receiving exogenous somatotropin. However, the increased serum IGF1 was associated with greater *IGF1* mRNA in the mammary fat pat due to estrogen applications. As suggested by these authors, *IGF1* mRNA expression may be more accentuated in mammary stromal tissue, which can explain the absence of statistical difference obtained in our study for *IGF1* response because our samples consisted of a minimum mammary fat pad. The limiting response in *ER1* could partially be explained by the same reason, as according to Connor et al. [87], *ER1* can be found on both epithelial cells and the mammary fat pad of prepubertal heifers.

Considering our overall findings, we associated the reduction in *IGFBP3* expression with the increased IGF1 serum concentration, which resulted in greater parenchyma ductal growth represented by the reduced pixel value in the mammary ultrasound of bST-treated heifers.

## Conclusions

The administration of bovine somatotropin in prepubertal Holstein × Gyr heifers fed for high daily gain rates does not improve growth parameters, feed efficiency, or final body weight. However, bST injections can increase the IGF1 serum concentration and, as a result, we can expect improved metabolism, mammary parenchyma growth, and lean carcass gain of heifers. In summary, we confirm the efficacy of bST injections to overcome the detrimental effects of high-gain diets on mammary gland growth and to improve lean carcass gain of prepubertal Holstein × Gyr heifers.

## Supporting information

S1 Table Serum progesterone and reproductive information. (XLSX)

S2 Table Analyses dataset. (XLSX)
